# Acceleration of bone-defect repair by using A-W MGC loaded with BMP2 and triple point-mutant HIF1α-expressing BMSCs

**DOI:** 10.1186/s13018-015-0219-3

**Published:** 2015-05-28

**Authors:** Yuzhong Gao, Chen Li, Hao Wang, Guangyu Fan

**Affiliations:** Department of Orthopedics, The Affiliated First Hospital of China Medical University, No. 155, Nanjing North Street, Heping District, 110001 Shenyang, Liaoning province China; 2nd Ward of Bone and Joint, The First Affiliated Hospital of Liaoning Medical University, No.2, Wuduan, Renmin Street, 121001 Jinzhou, China; Biobank, The First Affiliated Hospital of Liaoning Medical University, No.2, Wuduan, Renmin Street, 121001 Jinzhou, China

**Keywords:** Bone defects, Bone marrow mesenchymal stem cells, Bone morphogenetic protein 2, Hypoxia-inducible factor 1α

## Abstract

**Background:**

The goal of this study is to explore the effects of A-W MGC (apatite-wollastonite magnetic bioactive glass-ceramic) loaded with BMP2 (bone morphogenetic protein 2)- and HIF1α^mu^ (hypoxia-inducible factor 1 mutation)-expressing BMSCs (bone marrow mesenchymal stem cells) on the bone defect repair.

**Methods:**

(1) BMSCs were infected with viral solution containing BMP2 and HIF1α^mu^ with the best MOI (multiplicity of infection). The efficiency was observed via hrGFP (human renilla reniformis green fluorescent protein). (2) The cells were divided into five groups (A–E), and ALP (alkaline phosphatase) activity was measured. (3) BMP2 and HIF1α (hypoxia-inducible factor 1α) protein were measured. (4) A-W MGC was loaded with BMSCs that contain the genes and implanted into the bone defect model. The animals were sacrificed 8 and 12 weeks later. (5) The healing was measured with X-ray, histology, and biomechanics.

**Results:**

(1) BMSCs in A–D showed high transfection efficiency. (2) ALP in A and B was higher than the others (*p* = 0.041 or 0.038); A was higher than B (*p* = 0.038); (3) BMP2 in A and B was higher than the others (*p* = 0.014). HIF1α in A and C was higher than the others (*p* = 0.020). (4) 8 and 12 weeks after, an X-ray indicated that bone defect was nearly fully repaired in A and C. (5) 12 weeks after, the bone remodeling was complete in A and C. (6) The flexural strength in A and C was stronger than the others (*p* = 0.043).

**Conclusion:**

Engineered A-W MGC with BMP2 and HIF1α^mu^-expressing BMSCs exhibits comparable therapeutic effects of bone-defect repair as an autologous bone graft.

## Background

Treatment of large bone defects caused by trauma has been a major unsolved problem in the orthopedics field [1, 2]. Clinically, bone grafts have remained the standard treatment to restore the integrity and biomechanical properties of the bone defects. The breakthrough in creating a new type of engineered bone grafts, which combine the osteogenic factors, osteogenic stem cells, and the artificial bone scaffold, provides a new way for the bone-defect treatment.

BMP (bone morphogenetic protein) is one of the osteogenic factors that play important roles in the ontogenesis. BMP can stimulate the differentiation of mesenchymal cells into osteogenic cells and enhance osteoblasts [3], which initiates a series of bone regeneration steps to improve bone healing. However, due to the damage of the blood vessels of the microcirculation system at the injury site, it is difficult to rebuild a vascular system in the core part of the large engineered bone graft (diameter >5 mm), resulting in poor bone growth and the failure of bone graft transplantation. Meanwhile, the transfected osteogenic cells stop growing due to insufficient nutrient supply, resulting in the difficulty of new bone formation inside the engineered bone graft. Therefore, it is critical to build an effective network of blood vessels within the bone graft as early as possible and form sufficient connections with the blood vessels in the host bones to provide nutritional and metabolic supply and stimulate osteogenic differentiation, thereby promoting healing and repair of bone defects.

Angiogenesis is regulated by a series of growth factors [4–6]. Because HIF1α (hypoxia-inducible factor 1α) plays an important role in promoting angiogenesis, it is considered the most promising gene in the clinical application [7–11]. However, HIF1α can only be expressed and accumulated under hypoxic conditions. The 402 and 564 proline in the ODDD (oxygen-dependent degradation domain) in the CDS (coding sequence) cannot be hydroxylated, which causes the rapid degradation (within minutes) of the endogenous HIF1α [12–15]. Lando et al. have reported [16] that the transcriptional activity of HIF1α can be reduced by the function of the 803 asparagine in the CDS area, suggesting that the 402 and 564 proline and the 803 asparagine in the CDS area are important loci of HIF1α. In our previous research, we have created HIF1α^mu^ (hypoxia-inducible factor 1 mutation), which not only can be expressed under normoxic condition in vitro but also can promote angiogenesis in the bone-defect area under normoxic conditions in vivo [17]. In this current study, we constructed engineered bone graft using A-W MGC (apatite-wollastonite magnetic bioactive glass-ceramic) combined with BMSCs that are co-infected with adenovirus vectors containing HIF1α^mu^ and BMP2 genes. Our goal is to explore the interaction of HIF1α^mu^ and BMP2 and the impact on angiogenesis and ontogenesis in bone-defect area.

## Materials and methods

### Materials

The reagents used in this study are shown in Table 1.

**Table 1:** Reagents

Name	Company
Adv-HIF1α^mu^-hrGFP (1.6 × 10^8^ pfu/mL)	First Affiliated Hospital of Liaoning Medical Tissue Engineering Laboratory
Adv-BMP2-hrGFP (2.0 × 10^8^ pfu/mL)	First Affiliated Hospital of Liaoning Medical Tissue Engineering Laboratory
Rabbit anti-human HIF1α monoclonal antibody, rabbit anti-human BMP2 monoclonal antibody	
Secondary antibodies, β-actin	Santa Cruz, USA
DMEM (low glucose, catalog number: 10567-014), trypsin, fetal bovine serum	Gibco, USA
A-W MGC biological scaffolds	First Affiliated Hospital of Liaoning Medical Tissue Engineering Laboratory
ALP kit	TaKaRa, Japan
Other reagents	First Affiliated Hospital of Liaoning Medical Tissue Engineering Laboratory

*HIF1αmu* hypoxia-inducible factor 1 mutation, *hrGFP* human renilla reniformis green fluorescent protein, *BMP2* bone morphogenetic protein 2, *ALP* alkaline phosphatase, *A-W MGC* apatite-wollastonite magnetic bioactive glass-ceramic

### Animals

Sixty-one purebred New Zealand white rabbits, 3–4 months of age, weighing 2.0–2.8 kg, male or female, were provided by the Experimental Animal Center of Liaoning Medical University. All experimental procedures were carried out in accordance with the experimental animal and animal welfare requirements of Liaoning Medical Experiments Animal Ethics Committee.

## Methods

### Infection of BMSCs with recombinant adenovirus

The New Zealand white rabbit was deeply anesthetized, and the bone marrows from the right tibial bone were harvested. BMSCs were isolated, purified, and cultured to the third generation. The infections of Adv-HIF1α^mu^-hrGFP and Adv-BMP2-hrGFP were carried out with the best MOI (multiplicity of infection): MOI = 100 (HIF1α) and MOI = 150 (BMP2). The infection efficiency was evaluated with inverted fluorescence microscope 72 h after the infection.

### Experimental groups

- Group A: BMSCs were infected with viral solution containing Adv-BMP2-hrGFP and Adv-HIF1α^mu^-hrGFP.
- Group B: BMSCs were infected with viral solution containing Adv-BMP2-hrGFP.
- Group C: BMSCs were infected with viral solution containing Adv-HIF1α^mu^-hrGFP.
- Group D: BMSCs were infected with viral solution containing Adv-hrGFP.
- Group E: BMSCs without infection of any viral solution.

### Detection of the cell ALP activity and concentration

The supernatant in 96-well culture plates was removed 3, 6, 9, and 12 days after infection; the cells were washed once with PBS. The number of cells per well was estimated and adjusted to a similar value for each well. 100 μL 0.1 % Triton X-100 was added to each well and incubated at 4 °C overnight. All procedures were carried out according to the manufacturer’s instructions. The absorbance change per minute (ΔA/min) was measured at 405 nm and monitored for three consecutive minutes. The ALP (alkaline phosphatase) activity (U/L) = ΔA/min × 2757.

### Measurement of BMP2 and HIF1α protein expressions by Western blot

The total protein from each group was extracted using lysis buffer and the protein concentration was measured by BCA assay. Equal amounts of total protein from each group were separated by SDS-PAGE (5 and 8 %), 60 V × 30 min, 150 V × 1 h. After three washes, the proteins were transferred to PVDF membranes (100 mA × 30 min). The membranes were incubated with primary antibodies (1:1500) at 4 °C overnight. The membranes were washed and incubated with secondary antibodies and developing solution in a dark room for 30 min at room temperature. The membranes were washed three times with triple-distilled water to terminate the color reaction. The target bands on the membranes were analyzed by gel imaging system with the reference OD values. The experiment was repeated three times to calculate the relative OD values.

### Production of bone-defect animal model

#### Pre-operative treatment of biological scaffolds

A-W MGC biological scaffolds, 1.5 cm length, 0.5 cm diameter, were sterilized, surface prepared, and dried. Seventy-two hours after viral infection, the BMSCs were suspended at a density of 2 × 10^7^/mL and then transplanted into A-W MGCs, 5 × 10^6^ each side. The A-W MGCs were rocking cultured at 37 °C, 5 % CO_2_ for 48 h, and the cells were evenly and fully adhered to the internal space of the biological scaffolds.

#### Animal model of bone defects

New Zealand white rabbits were anesthetized and a 1.5-cm segment from the middle section of the right radius was removed to create a bone-defect region. The pre-treated A-W MGCs were anastomosed end-to-end to the stumps of the broken bones. After the surgery, the animals were given intramuscular injection of antibiotics for 7 days. The animals’ daily activities and wound healing process were closely monitored. Animals were sacrificed 8 and 12 weeks after the surgery. All animal models were produced by the same operator.

#### Experimental groups

Sixty New Zealand white rabbits were randomly divided into A, B, C, D, and E groups of 12.

- Group A: A-W MGC transplanted with BMSCs that were infected with Adv-HIF1α^mu^-hrGFP and Adv-BMP2-hrGFP.
- Group B: A-W MGC transplanted with BMSCs that were infected with Adv-BMP2-hrGFP.
- Group C: autogenous bone graft group (contralateral radial transplantation).
- Group D: A-W MGC transplanted with BMSCs that were infected with Adv-HIF1α^mu^-hrGFP.
- Group E: A-W MGC scaffold alone.

### X-ray observation

Eight or 12 weeks after surgery, the animals were anesthetized and the x-ray images of the radius and ulna at the ipsilateral site of surgery were taken. Projection conditions were 40 kV, 50 mA, 0.2 s, and a projection distance of 60 cm.

### Histological examination

The animals in each group were sacrificed 12 weeks after the surgery. The radius and ulna at the bone-defect area were harvested. The degradation of the implant graft, the functional connection between the graft and the host bone, and the surrounding soft tissue response were observed. The tissues from the center of the bone-defect area were decalcified in 5 % nitric acid for 72 h, fixed in 4 % formalin, paraffin-embedded, and sliced continuously with a thickness of 10 μm. HE staining was performed.

### Biomechanical measurement

Twelve weeks after the surgery, the samples from each group were tested with a universal testing machine (AG-IC, SHIMADZU) for anti-flexion strength with a span of 30 mm and a loading rate of 0.5 mm/min. The flexural strength of new bones at the bone-defect area in each group was compared.

### Statistical analysis

SPSS 18.0 for Windows software package was used for statistical analysis. Data were expressed as mean ± standard deviation (*x ± s*). One-way ANOVA was used for the comparisons among groups (normally distributed data). Non-parametric statistics (Wilcoxon rank-sum test) were used for analyzing non-normally distributed data. *p* < 0.05 was considered statistically significant.

## Results

### BMSCs were successfully infected with recombinant adenovirus

**Fig. 1:**
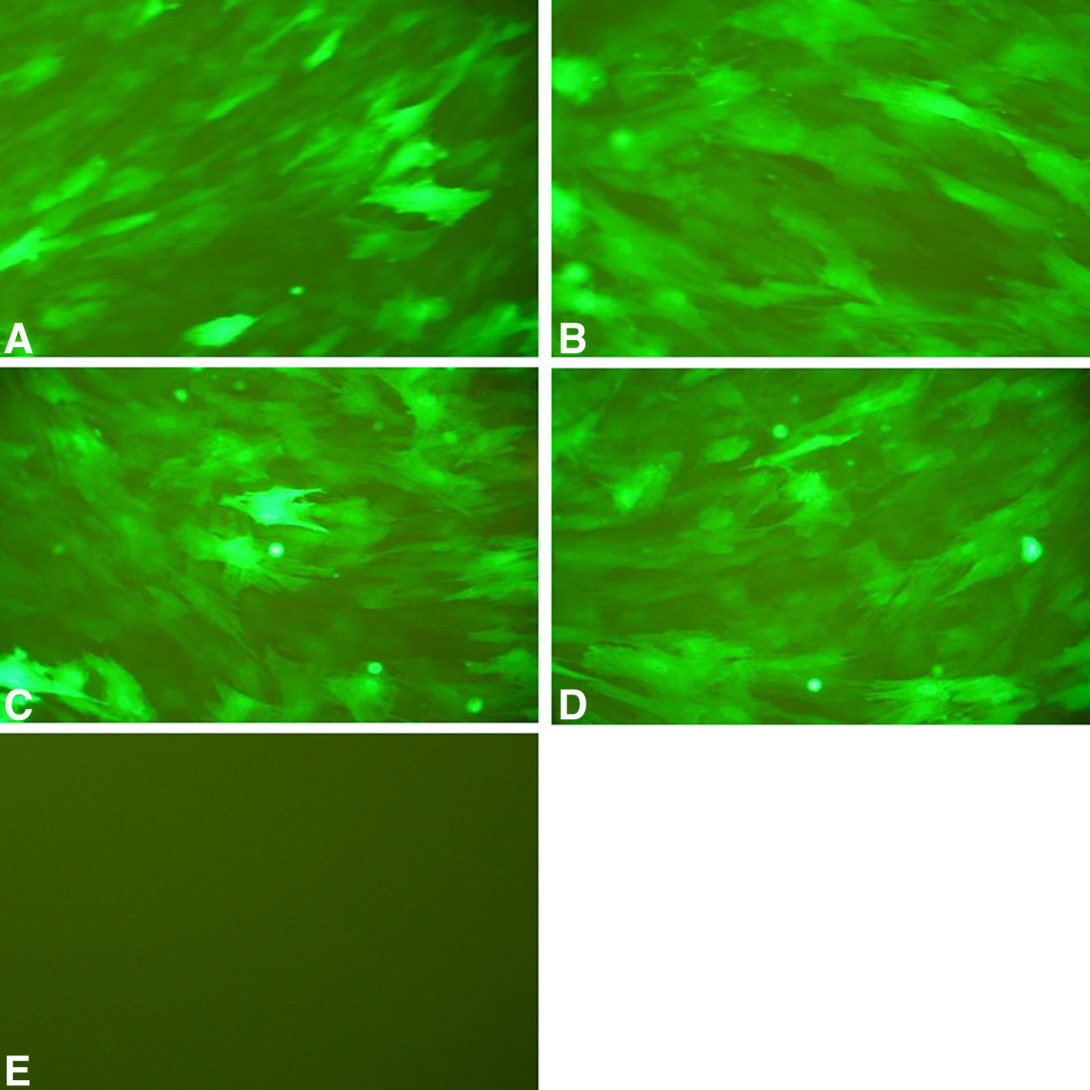
The green fluorescence expressions in each group (×200). **a** BMSCs that were infected with Adv-BMP2-hrGFP and Adv-HIF1α^mu^-hrGFP. **b** BMSCs that were infected with Adv-BMP2-hrGFP. **c** BMSCs that were infected with Adv-HIF1α^mu^-hrGFP. **d** BMSCs that were infected with Adv-hrGFP. **e** BMSCs without infection. BMSCs: mesenchymal stem cells. BMP2: bone morphogenetic protein 2. hrGFP: human renilla reniformis green fluorescent protein. HIF1α^mu^: hypoxia-inducible factor 1 mutation

The BMSCs in groups A, B, C, and D showed strong expressions of green fluorescent protein under a fluorescence microscope 72 h after infection. The number of BMSCs expressing green fluorescent protein was high, indicating the efficient infection rate (Fig. 1).

### ALP activity in each cell group

**Fig. 2:**
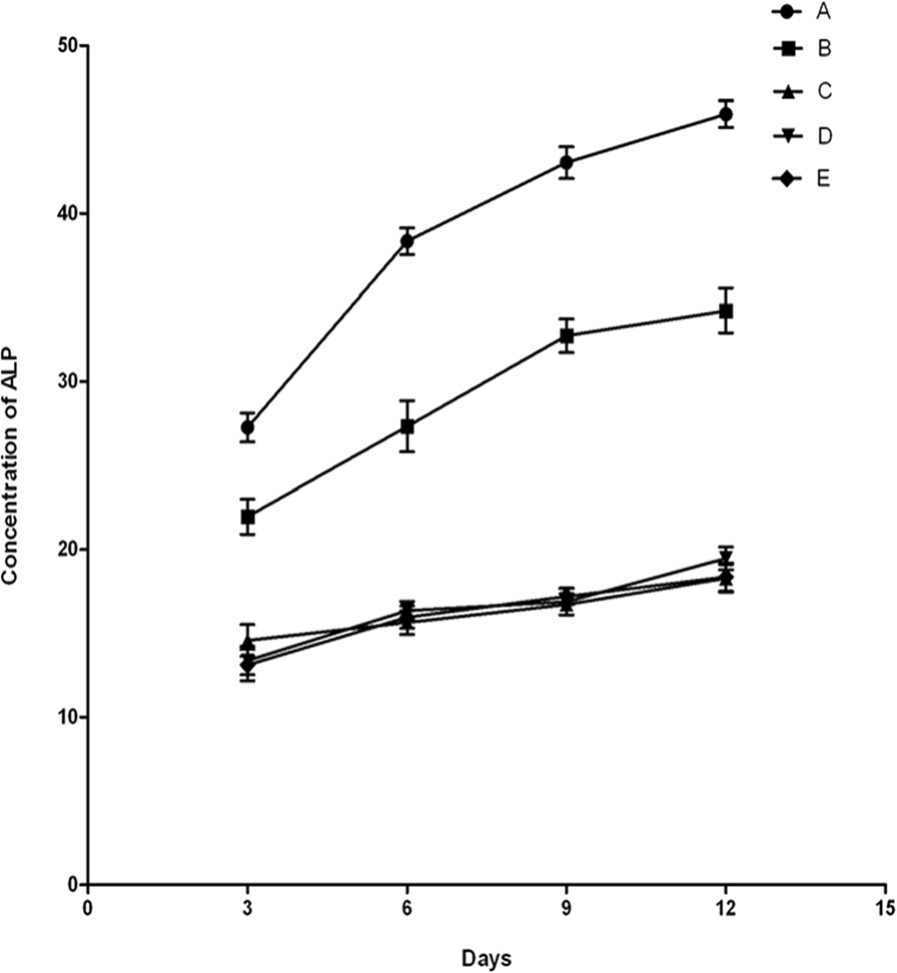
The ALP concentration at different time points in each BMSC group. **a** BMSCs that were infected with Adv-BMP2-hrGFP and Adv-HIF1α^mu^-hrGFP. **b** BMSCs that were infected with Adv-BMP2-hrGFP. **c** BMSCs that were infected with Adv-HIF1α^mu^-hrGFP. **d** BMSCs that were infected with Adv-hrGFP. **e** Blank control BMSCs (no infection). ALP: alkaline phosphatase. BMSCs: mesenchymal stem cells. BMP2: bone morphogenetic protein 2. hrGFP: human renilla reniformis green fluorescent protein. HIF1α^mu^: hypoxia-inducible factor 1 mutation

ALP activity was detected according to the manufacturer’s instructions. The absorbance change per minute (ΔA/min) was measured at 405 nm and monitored for three consecutive minutes. The ALP activity (U/L) = ΔA/min × 2757. The ALP in group A was significantly higher than the B, C, D, and E groups (*p* = 0.038). The ALP in group B was significantly higher than the C, D, and E groups (*p* = 0.041). There was no significant difference among C, D, and E groups (*p* = 0.926) (Fig. 2 and Table 2).

**Table 2:** The measurement of ALP in each BMSC group at different time points (U/L, *N* = 8, x ± s)

Group	3 days	6 days	9 days	12 days
A	28.74 ± 1.37	37.89 ± 1.73	42.87 ± 2.38	45.12 ± 2.22
B	21.18 ± 1.34*	26.91 ± 3.62*	32.12 ± 2.41*	33.79 ± 2.57*
C	14.27 ± 0.73*#	15.94 ± 1.26*#	16.36 ± 1.28*#	17.15 ± 0.74*#
D	12.99 ± 0.62*#	16.39 ± 0.95*#	16.71 ± 1.08*#	18.01 ± 1.24*#
E	13.38 ± 0.85*#	16.21 ± 1.08*#	16.54 ± 1.23*#	17.08 ± 0.91*#

*ALP* alkaline phosphatase, *BMSCs* bone marrow mesenchymal stem cells

*Compared with group A, *p* < 0.05; #compared with group B, *p* < 0.05

### BMP2 and HIF1α protein expressions in each BMSCs group

**Fig. 3:**
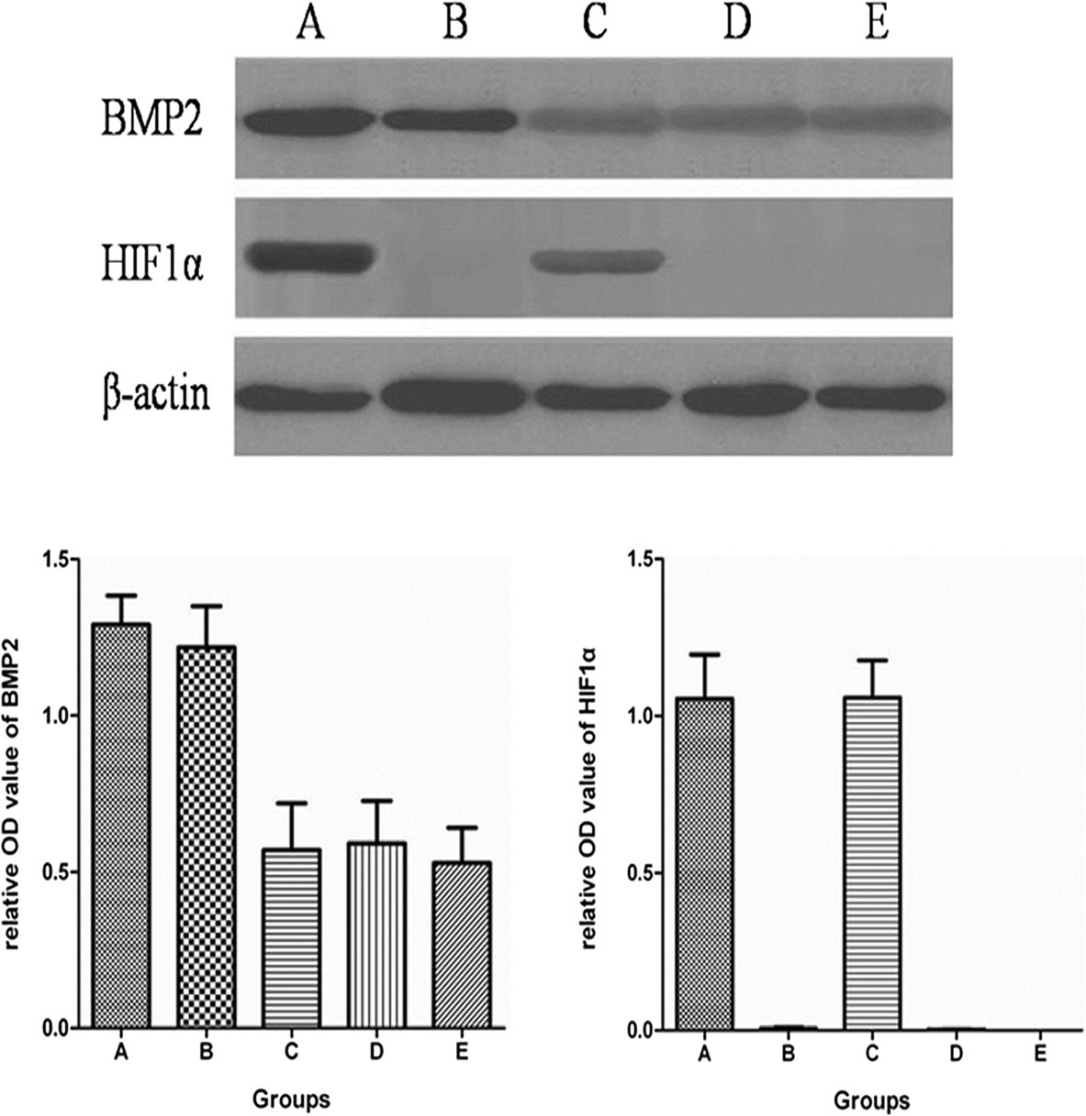
The BMP2 and HIF1α protein expressions in the five BMSC groups. **a** BMSCs that were infected with Adv-BMP2-hrGFPand Adv-HIF1α^mu^-hrGFP. **b** BMSCs that were infected with Adv-BMP2-hrGFP. **c** BMSCs that were infected with Adv-HIF1α^mu^-hrGFP. **d** BMSCs that were infected with Adv-hrGFP. **e** Blank control BMSCs (no infection). BMSCs: mesenchymal stem cells. BMP2: bone morphogenetic protein 2. hrGFP: human renilla reniformis green fluorescent protein. HIF1α^mu^: hypoxia-inducible factor 1 mutation

The BMP2 protein expression was significantly higher in the A and B groups, compared with groups C, D, and E (*p* = 0.014). The BMP2 protein expression was slightly higher in group A than group B; however, the difference was not significant (*p* = 0.069). The HIF1α protein expression was significantly higher in the A and C groups, compared with groups B, D, and E (*p* = 0.020). There was no significant difference among groups B, D, and E, indicating that mutant HIF1α can express functional proteins under normoxic conditions, whereas wild type HIF1α cannot exist under normoxic conditions (Fig. 3).

### X-ray examination

**Fig. 4:**
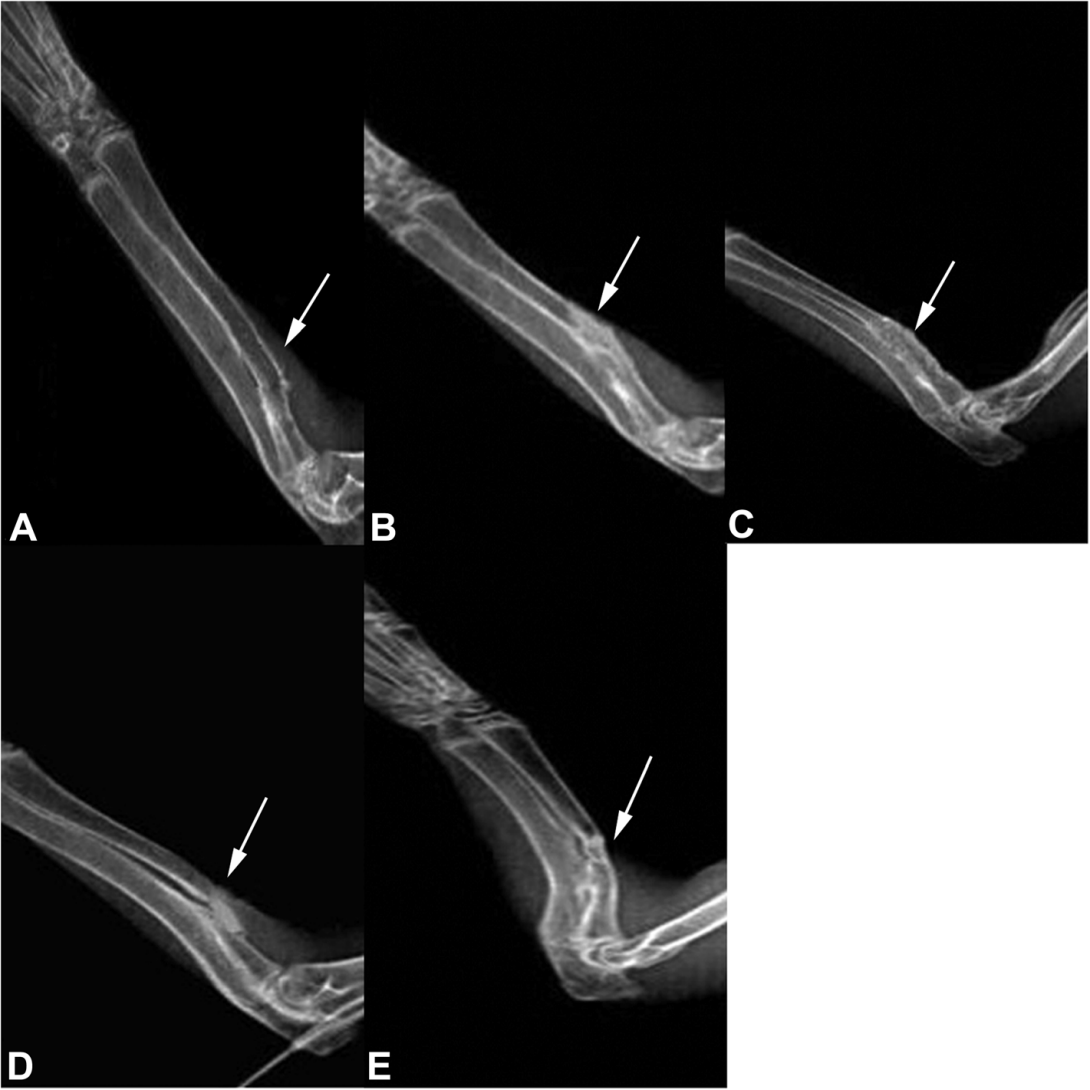
X-ray showed the bone-defect areas in different groups at 8 weeks after the surgery. **a** A-W MGC transplanted with BMSCs that were infected with Adv-HIF1αmu-hrGFP and Adv-BMP2-hrGFP. **b** A-W MGC transplanted with BMSCs that were infected Adv-BMP2-hrGFP. **c** autogenous bone graft group. **d** A-W MGC transplanted with BMSCs that were infected with Adv-HIF1α^mu^-hrGFP. **e** A-W MGC. *Arrows*: defect sites. MGC: magnetic bioactive glass-ceramic. BMSCs: mesenchymal stem cells. BMP2: bone morphogenetic protein 2. hrGFP: human renilla reniformis green fluorescent protein. HIF1α^mu^: hypoxia-inducible factor 1 mutation

**Fig. 5:**
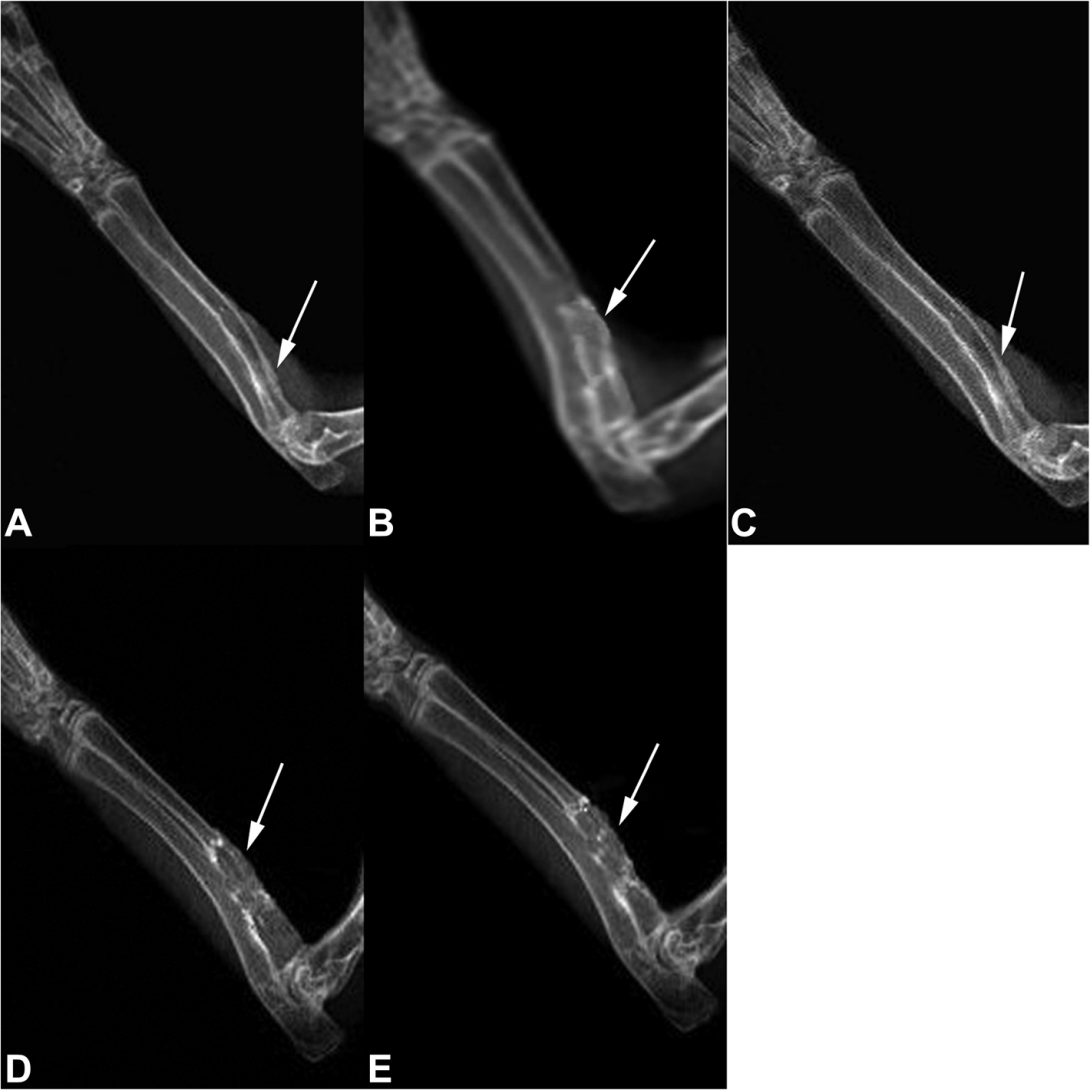
X-ray showed the bone-defect areas in different groups at 12 weeks after the surgery. **a** A-W MGC transplanted with BMSCs that were infected with Adv-HIF1αmu-hrGFP and Adv-BMP2-hrGFP. **b** A-W MGC transplanted with BMSCs that were infected Adv-BMP2-hrGFP. **c** autogenous bone graft group. **d** A-W MGC transplanted with BMSCs that were infected with Adv-HIF1α^mu^-hrGFP. **e** A-W MGC. *Arrows*: defect sites. MGC: magnetic bioactive glass-ceramic. BMSCs: mesenchymal stem cells. BMP2: bone morphogenetic protein 2. hrGFP: human renilla reniformis green fluorescent protein. HIF1α^mu^: hypoxia-inducible factor 1 mutation

Eight weeks after the surgery, the bone intensity in the bone-defect area in group A (double gene infection group) and group C (autogenous bone graft group) significantly increased, similar to the normal bone tissue; the fracture line was barely visible, and bone defect was completely reconnected. In group B, the new bone callus increased, but the fracture line at both ends was still visible and the bone defect has not been fully reconnected. In group D, there was a small amount of callus formation, but the bone defect has not been fully reconnected and the fracture line at both ends was clearly visible. In group E, the implant material and the fracture line were clearly visible without signs of absorption of the implant material (Fig. 4). Twelve weeks after the surgery, the bone defect was completely repaired in group A and group C, the bone structure was improved, and the bone density was similar to the normal bone tissue. The cortical and medullary cavity structures were visible. In group B, the bone density in the defect area was significantly increased. In group D, the initial bone-defect repair and remodeling were slow. In group E, there was sclerosis, with no bone-defect repair or visible bone marrow cavity (Fig. 5).

### Histological observation

**Fig. 6:**
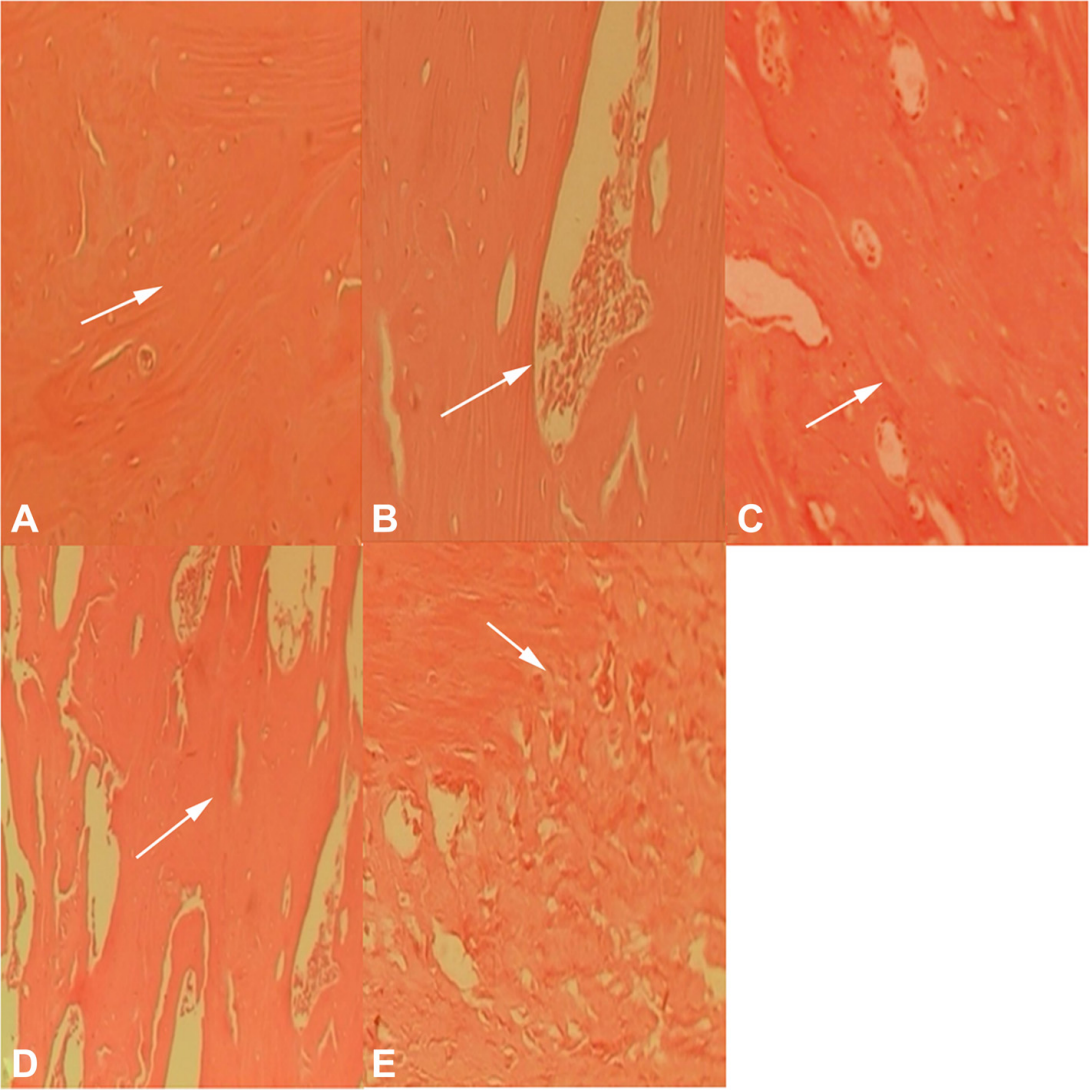
HE staining images (×100) from different groups 12 weeks after surgery. **a** A-W MGC transplanted with BMSCs that were infected with Adv-HIF1αmu-hrGFP and Adv-BMP2-hrGFP. **b** A-W MGC transplanted with BMSCs that were infected Adv-BMP2-hrGFP. **c** Autogenous bone graft group. **d** A-W MGC transplanted with BMSCs that were infected with Adv-HIF1α^mu^-hrGFP. **e** A-W MGC. *Arrows*: cell nuclei. HE: hematoxylin and eosin. A-W MGC: apatite-wollastonite magnetic bioactive glass-ceramic. BMSCs: mesenchymal stem cells. BMP2: bone morphogenetic protein 2. hrGFP: human renilla reniformis green fluorescent protein. HIF1α^mu^: hypoxia-inducible factor 1 mutation

Twelve weeks after the surgery, the A-W MGC graft was completely degraded in group A. The new bone tissues showed mature bone structure and visible trabeculae. In group B, the bone defect was reconnected, but there was still residual carrier scaffold indicating the degradation was incomplete. In group C, the cortex and medulla of the bone defect were completely reconnected. The bone remodeling and healing were complete. In group D, there were less trabecular bones and the carrier material was not absorbed. In group E, there was clear bone defect, poor reconnection, and visible fibrous connective tissue filling (Fig. 6).

### Biomechanical test (flexural strength test)

**Fig. 7:**
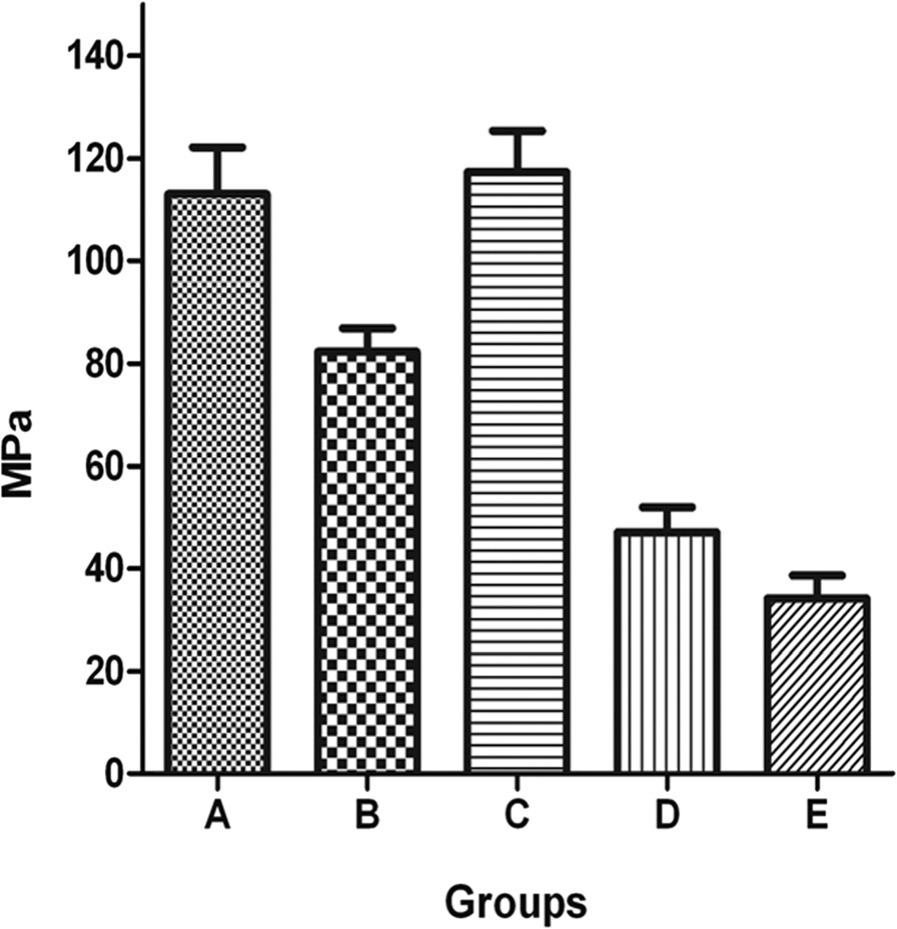
The results of flexural strength test from each group. **a** A-W MGC transplanted with BMSCs that were infected with Adv-HIF1αmu-hrGFP and Adv-BMP2-hrGFP. **b** A-W MGC transplanted with BMSCs that were infected Adv-BMP2-hrGFP. **c** Autogenous bone graft group. **d** A-W MGC transplanted with BMSCs that were infected with Adv-HIF1αmu-hrGFP. **e** A-W MGC. A-W MGC: apatite-wollastonite magnetic bioactive glass-ceramic. BMSCs: mesenchymal stem cells. BMP2: bone morphogenetic protein 2. hrGFP: human renilla reniformis green fluorescent protein. HIF1α^mu^: hypoxia-inducible factor 1 mutation

The results of flexural strength test from each group were as follows: group A, 107.59 ± 4.91 MPa; group B, 82.34 ± 5.03 MPa; group C, 112.94 ± 6.08 MPa; group D, 52.41 ± 4.26 MPa; group E, 33.27 ± 4.55 MPa. There was no significant difference between A and C groups (*p* = 0.88). There were significant differences between all the other two-group comparisons (*p* = 0.043) (Fig. 7).

## Discussion

In this study, we used A-W MGC scaffold loaded with BMSCs that were infected with BMP2 and HIF1α^mu^ genes for the treatment of in vivo bone defects and achieved therapeutic effects that were comparable with an autogenous bone graft. Firstly, the successful reestablishment of microcirculation system at the bone-defect site not only provides osteoblasts with nutrition but also has anti-apoptotic effects. Therefore, it will promote the stable growth of osteoblasts, accelerate bone healing process, and greatly improve the survival rate of transplanted bone graft [18–20]. Secondly, a single factor may not be able to sufficiently repair the damages to the systems caused by the bone defect; in this case, it might be more sufficient to apply multiple genes to achieve individualized repair on different systems. Therefore, this experiment not only provides the foundation for the treatment of bone defects using multigene therapy, but also offers new ideas to broaden the gene therapy in other ischemic diseases.

### HIF1α and BMP2 as the target genes

The key point in the gene therapy of bone defects is to introduce the osteogenic factors into the seed cells used for the tissue engineered bone graft. These seed cells can continue to proliferate in vivo and secret growth factors to promote fracture healing [21]. Among these osteogenic factors, BMP2 has the most significant effect on the bone regeneration [22–24]. Therefore, we used an adenovirus vector carrying BMP2 gene to infect BMSCs in order to achieve a sustained localized expression of bone morphogenetic protein to accelerate fracture healing.

Local application of angiogenic factors has been a part of the adjuvant treatment in bone-defect diseases and has not received much attention for the past years. However, people start to realize that it is equally important to establish a local blood circulation to prevent the death of osteoblasts after transplantation. HIF1α is a transcription factor generated under hypoxic conditions and is sensitive in the detection of local tissue hypoxia. The concentration of HIF1α increases with prolonged hypoxia and decreases rapidly after the improvement of hypoxia [25]. Under the temporary anoxic conditions, HIF1α not only can maintain the survival of osteoblasts but also can neutralize the harmful substances produced by hypoxic cells, creating a favorable osteogenic microenvironment [25]. Therefore, HIF1α is more efficient in promoting angiogenesis than VEGF (vascular endothelial growth factor). To date, no report has been found on the effects of HIF1α combined with BMP2 multigene therapy in bone-defect diseases. HIF1α cannot stably express under normoxic conditions. To overcome this shortcoming, we have successfully constructed a triple mutant HIF1α by point mutation of the 402,564 and 803 amino acids without affecting other functional areas. The triple point-mutant HIF1α can express functional protein under normoxic conditions and play important physiological roles in angiogenesis [17].

### The interaction between HIF1α and BMP2

The increase of ALP concentration is a specific marker indicating the differentiation of BMSCs into osteoblasts. In order to analyze the impact of HIF1α^mu^ on BMP2 expression, we measured the ALP from BMSCs that were infected with HIF1α^mu^ plus BMP2, the BMSCs that were infected with only BMP2, and the BMSCs without infection at 3, 6, 9, and 12 days after infection. We found that the ALP activity significantly increased in the BMSCs that were infected either with HIF1α^mu^ plus BMP2 or with only BMP2, compared with uninfected BMSCs. Compared with the BMSCs that were infected with only BMP2, the ALP activity in the BMSCs that were infected with HIF1α^mu^ plus BMP2 was significantly higher (*p* < 0.05), suggesting that HIF1α^mu^ can promote BMP2 expression and increase ALP level.

### The advantages of A-W MGC as a scaffold

An ideal artificial scaffold should have the following characteristics [26]: 1) has good biocompatibility (without inflammation with host tissue) and is biodegradable; 2) has excellent osteoinductive and osteoconductive effects; and 3) can provide good osteogenic environment to support the growth of seed cells. A-W MGC scaffold used in this experiment was made of bioactive ceramics and has a stronger cell adhesion by a surface treatment of the chitosan. A-W MGC has a large number of 2–3 μm pores, and these well-interconnected pores favor the cell adhesion and proliferation, promote angiogenesis and blood vessel network formation, and accelerate the merge of new bone and bio-material. Therefore, A-W MGC exhibits good biocompatibility, osteoconductive/osteoinductive effects, and degradability [27], meets the requirements for bone tissue engineering material, and becomes the preferred carrier scaffold.

### The new tissue engineered bone graft achieved comparable therapeutic effects as the autologous bone graft

X-ray results indicate that the bone formation and bone remodeling capabilities in group A (A-W MGC seeded with BMSCs that were infected with HIF1α^mu^ plus BMP-2) were similar with group C (autogenous bone graft group) (*p* > 0.05). Meanwhile, the bone formation and bone remodeling capabilities in A and C groups were superior to the other groups.

In this study, HE staining was used to observe the degradation of the implant materials, the graft-host bone interface, and the surrounding soft tissue reactions. Histological results indicate that A-W MGC in group A was fully degraded 12 weeks after surgery and the new mature bone tissues and trabecular bones were more visible. The healing of bone defect was similar compared with group C, whereas the osteogenic effect was not very obvious in the other three groups.

Flexural strength test is the most convincing indicator for the mechanical properties of bone formation. The change in the morphology and mechanical properties of bone tissue by the external force is a reliable method for the evaluation of osteogenic effects. The results from biomechanical testing 12 weeks after surgery indicate that the engineered bone graft in group A had similar effects on the bone regeneration in the bone-defect area compared with autogenous bone graft, whereas the osteogenic effect from the other groups were much less effective.

## Conclusion

In the present study, we constructed A-W MGC bone graft loaded with BMSCs that were infected with both HIF1α^mu^ and BMP2. The angiogenic effects of HIF1α^mu^ and the osteogenic effects of BMP2 interacted with each other, promoted the healing process of the bone defect, and achieved similar results as autologous bone graft. These results indicate that this A-W MGC bone graft will be an ideal alternative for autologous bone graft and solve the problems due to the insufficient number of autologous bone grafts and the complications of autologous bone transplantation. However, angiogenesis and ontogenesis are extremely complex physiological processes. The introduction of the exogenous triple point-mutant HIF1α and BMP2 genes promotes the formation of blood vessel network and bone regeneration; however, the interaction of these exogenous genes with the endogenous angiogenic and osteogenic factors is still unknown. The impact of the mutation on the function of HIF1α itself and BMP2, as well as the function of the three alanine mutations in the angiogenic process, needs to be further explored in our future studies.

## Abbreviations

A-W MGC: Apatite-wollastonite magnetic bioactive glass-ceramic
BMP2: Bone morphogenetic protein 2
HIF1α^mu^: Hypoxia-inducible factor 1 mutation
BMSCs: Mesenchymal stem cells
MOI: Multiplicity of infection
hrGFP: Human renilla reniformis green fluorescent protein
ALP: Alkaline phosphatase
BMP: Bone morphogenetic protein
HIF1α: Hypoxia-inducible factor 1α
ODDD: Oxygen-dependent degradation domain
CDS: Coding sequence
VEGF: Vascular endothelial growth factor
